# Performance of *Treponema pallidum* recombinant proteins in the serological diagnosis of syphilis

**DOI:** 10.1371/journal.pone.0234043

**Published:** 2020-06-18

**Authors:** Ângelo Antônio Oliveira Silva, Ueriton Dias de Oliveira, Larissa de Carvalho Medrado Vasconcelos, Leonardo Foti, Leonardo Maia Leony, Ramona Tavares Daltro, Amanda Leitolis, Fernanda Washington de Mendonça Lima, Marco Aurélio Krieger, Nilson Ivo Tonin Zanchin, Fred Luciano Neves Santos

**Affiliations:** 1 Gonçalo Moniz Institute, Oswaldo Cruz Foundation (Fiocruz-BA), Salvador, Bahia, Brazil; 2 Federal University of Paraná, Curitiba, Paraná, Brazil; 3 Carlos Chagas Institute, Oswaldo Cruz Foundation (Fiocruz-PR), Curitiba, Paraná, Brazil; 4 Federal University of Bahia, Salvador, Bahia, Brazil; 5 Molecular Biology Institute of Paraná, Curitiba, Paraná, Brazil; Instituto Butantan, BRAZIL

## Abstract

Syphilis serodiagnosis is challenging because distinct clinical forms of the infection may influence serological performance and discordant results between tests make clinical decisions difficult. Several recombinant *Treponema pallidum*-proteins have already been tested for syphilis diagnosis and they are critical to achieve high accuracy in serological testing. Our aim was to assess the varied from performance of *T*. *pallidum*-recombinant proteins TmpA, TpN17 and TpN47 for syphilis serodiagnosis. The proteins were evaluated using sera of 338 *T*. *pallidum*-negative, 173 *T*. *pallidum*-positive individuals and 209 sera from individuals infected with unrelated diseases. The diagnostic potential was validated by analysis of ROC curves. In the liquid microarray analyses, the ROC curve varied from 99.0% for TmpA and TpN17 to 100% for TpN47. The sensitivity score yielded values of up to 90% for TpN17, 100% for TpN47 and 80.0% for TmpA. The lowest and highest specificity values were presented by TpN47 (91.9%) and TmpA antigens (100%), respectively. TpN47 showed the highest accuracy score (95.5%) among all the recombinant proteins assayed. For the ELISA, the ROC curve was 97.2%, 91.8% and 81.6% for TpN17, TmpA and TpN47, respectively. TpN17 and TmpA yielded a sensitivity of 69.9%, while TpN47 obtained a value of 53.8%. Specificity was almost 100% for all three proteins. No cross-reaction was observed for TpN17 in the serum samples from non-bacterial infections. Regarding leptospirosis-positive samples, cross-reactivity score was varied from 8.6 to 34.6%. This is most probably due to conservation of the epitopes in these proteins across bacteria. The use of recombinant proteins in immunoassays for syphilis diagnosis was showed provide greater reliability to results of the treponemal assays. Despite the low sensitivity, the proteins showed high diagnostic capacity due to the AUC values found. However, an improvement in sensitivity could be achieved when antigenic mixtures are evaluated.

## Introduction

Syphilis is a human multisystemic infection caused by a spirochete bacterium called *Treponema pallidum*, subspecies *pallidum* (order Spirochaetales). Most cases of syphilis are due to sexual contact, hence the infection is considered a sexually transmitted infectious disease–STI [1]. Congenital syphilis occurs when pregnant women are infected and remains prevalent in many parts of the world [2]. It has been reported that cases of infection are also acquired through blood transfusion, needle sharing, contact with open lesions, organ transplantation, or occupational and other exposures [3–6]. Despite the existence of effective antibiotic therapy, the global burden of syphilis infection has increased drastically throughout the world in the last decades, with an estimated 10.6 million cases yearly, becoming a global health concern [7].

After initial contact with skin or mucous membranes, spirochetes replicate locally eliciting an inflammatory response and disseminating through blood vessels and lymphatics [8]. A distinctive painless, usually solitary, clean-based, indurated ulcer (chancre) typically appears three weeks after exposure. In penicillin-treated individuals, the ulcer begins to resolve within a few days, while in untreated individuals, primary lesions spontaneously resolve without scarring within 3–6 weeks. By this time, spirochetes disseminate from the primary site of infection to several organ tissues, mainly the skin, setting a new stage known as secondary syphilis [9]. This stage presents a broad range of mucocutaneous manifestations as well as systemic signs and symptoms within 4–10 weeks of the initial contact with *T*. *pallidum*. A robust cellular and humoral immune responses are present in an individual with secondary syphilis; however, one-third of untreated individuals develop potentially devastating forms of recrudescent disease referred to as tertiary syphilis, characterized as neurosyphilis, cardiovascular syphilis, or gummatous cutaneous syphilis [9].

Laboratory diagnosis of syphilis includes treponemal and non-treponemal tests. Non-treponemal tests, such as the VDRL (venereal disease research laboratory) and RPR (rapid plasma reagin), are employed to screen the infection and to monitor the therapeutic effect. Usually, they are easy-to-use, inexpensive and widely available; but further confirmatory screening tests are required for the detection of IgG anti-*T*. *pallidum*- antibodies by treponemal methods, including chemiluminescence, *T*. *pallidum* particle agglutination (TPPA) assay, *T*. *pallidum* hemagglutination (TPHA) assay, fluorescent treponemal antibody absorption test (FTA-ABS), enzyme-linked immunosorbent assay (ELISA) and immunochromatography. Nevertheless, these tests are more expensive and labor-intensive compared to non-treponemal tests [10]. Furthermore, some of them are highly operator-dependent. Among treponemal tests, ELISA is the most commonly used in syphilis diagnosis due to its simplicity, low cost and ease of automation. In addition, ELISA presents usually higher diagnostic performance compared to non-treponemal assays [11]. However, its performance depends on the antigens employed to detect the anti-*T*. *pallidum* antibodies presence and on the clinical stage of the infection [12].

Despite the existence of serological tests, the diagnosis of syphilis is still a challenge. Since there is no *T*. *pallidum* culture in axenic medium, serological tests are used as final diagnosis. Additionally, the distinct clinical forms of the infection may influence serological performance [13]. Therefore, clinical decisions have been impacted when serum samples from patients are confirmed as treponemal test reactive, yet non-treponemal nonreactive [14]. Several recombinant and native *T*. *pallidum*-proteins have already been tested as antigens in diagnostic tests for syphilis. Promising results were found using various diagnostic platforms [15–25]. However, there is no consensus on which antigens have the best serodiagnostic performance for syphilis. Accordingly, evaluation of recombinant *T*. *pallidum*-antigens is essential to develop accurate serological diagnostic tests for syphilis. Therefore, our aim was to assess the performance of *T*. *pallidum*-recombinant proteins TmpA, TpN17, TpN47 and TpN15 for syphilis serodiagnosis using distinct tools to antigen-antibody detection.

## Materials and methods

### Ethical statements

This study was approved by the Institutional Review Board (IRB) for Human Research at the Gonçalo Moniz Institute of the Oswaldo Cruz Foundation (IGM-FIOCRUZ), Salvador, Bahia-Brazil (CAAE: 67809417.0.0000.0040). To protect patient privacy, the IRB required that all samples be coded to mask patient identification, thereby avoiding the need for verbal or written consent. The data of all patients were fully anonymized before the researchers accessed the human sera samples.

### Recombinant proteins synthesis

Optimized synthetic genes for *E*. *coli* expression of *T*. *pallidum* subsp. *pallidum* Nichols strain were acquired from a commercial supplier (GenScript, Piscataway-NJ, USA). The synthetic genes purchased in pUC57 were subcloned in-house into the pET28a expression vector. Expression of the recombinant proteins was performed in *E*. *coli* strain BL21-Star (DE3) [26]. For this purpose, bacterial cells transformed with the respective expression vectors were first incubated for 16 h at 37°C in Luria-Bertani broth containing kanamycin (50 μg/ml). The culture was then diluted at 1:20 in fresh medium and reincubated at 37°C until an optical density ranging from 0.6–0.8 was attained, measured at 600 nm (OD_600_). Expression was induced by adding IPTG (isopropyl β-D-1-thiogalactopyranoside) to a final concentration of 500 μM and incubating for 4 h at 37°C. Bacterial cell disruption was performed using either a microfluidizing processor (Microfluidics Model M-110L, Hyland Scientific, Stanwood-WA, USA) or by chemical methods, and the resulting recombinant proteins were purified by affinity and ion-exchange chromatography. Proteins were quantified by fluorometric assay (Qubit12.0, Invitrogen Technologies, Carlsbad-CA, USA) and purity was verified by SDS-PAGE stained with CBB-G250 [27].

### Sampling

The human sera used in this study were divided into two panels. Panel 1 consisted of 338 *T*. *pallidum*-negative and 173 *T*. *pallidum*-positive samples obtained from the panel of the Blood Bank Screening and Hemotherapy Quality Control Program (AEQ-MS; Fiocruz/RJ, Brazil) (28), Pernambuco State Blood Bank (HEMOPE), Bahia State Blood Bank (HEMOBA), Clinical and Toxicological Analysis Laboratory of the Faculty of Pharmacy of Federal University of Bahia (LACTFAR-UFBA), Federal University of Goiás (UFG), Central Laboratory of Pernambuco (LACEN/PE) and Aggeu Magalhães Institute (FIOCRUZ/PE). Samples from the state of Bahia were acquired from December 2017 to April 2019, and from the states of Goiás and Pernambuco from June 2014 to December 2016. Initially, the ability of the recombinant antigens to distinguish *T*. *pallidum*-positive from negative samples was evaluated by liquid microarray (LMA) using the well-characterized sample set from the AEQ Panel. The antigens showing the highest diagnostic performance by LMA were then assayed by ELISA using samples from *T*. *pallidum*-infected and non-infected individuals living in the states of Bahia and Pernambuco. Panel 2, containing 209 samples from individuals infected with unrelated diseases previously diagnosed by parasitological or serological methods, was employed to evaluate cross-reactivity. This panel included chronic Chagas disease (n = 10), dengue (n = 10), filariasis (n = 10), hepatitis B virus (n = 20), hepatitis C virus (n = 20), HIV-1/2 (n = 20), HTLV-1/2 (n = 20), *Leishmania* spp. (n = 09) and leptospirosis (n = 80). All serum samples were serologically retested for non-specific *T*. *pallidum* antibodies using the RPR-BRAS (Laborclin LTDA, Curitiba-PR, Brazil), Immutrep^®^ USR Antigen (Omega Diagnostics LTD., Alva-Scotland, United Kingdom) assays or anti-*Treponema pallidum* IIFT (IgG) test (Euroimmun Medizinische Labordiagnostika AG, Lübeck, Germany). All testing was strictly performed according to manufacturer instructions. Any samples that returned discordant or inconclusive results were excluded. A unique identifier code was assigned to each sample to ensure a blinded analysis.

### Laboratory assays

The ability of recombinant antigens to efficiently differentiate between *T*. *pallidum*-positive and negative samples was evaluated by LMA, using a well-characterized set of samples obtained from the AEQ panel [28], and by ELISA, using samples of *T*. *pallidum*-infected and non-infected sera obtained from the HEMOBA, HEMOPE, LACTFAR-UFBA, UFG, LACEN/PE and FIOCRUZ/PE.

#### Liquid microarray

Coupling of *T*. *pallidum* antigens to paramagnetic carboxylated microspheres (Luminex Corp, Austin-TX, USA) was performed using the manufacturer’s protocol. Briefly, a suspension of 1 x 10^6^ microspheres was sonicated in an ultrasound bath (Cole-Parmer ultrasonic cleaner, Cole-Parmer Instruments Company, Vernon Hills-IL, USA) and horizontal agitation (IKA vortex genius 3 VG3S32, IKA do Brasil, Campinas-SP, Brazil) to ensure homogeneous distribution of the suspension. After two washes, the microspheres were suspended in 400 μl of activation buffer (100 mM sodium phosphate, pH 6.3). Solutions (50 μl of each) of N-hydroxysulfosuccinimide (Pierce, Rockford-IL, USA) and 1-ethyl-3(3-dimethylaminopropyl)-carbodiimide hydrochloride (Pierce), both diluted to 50 mg/ml in double-distilled water (dH_2_O), were added to chemically activate the microspheres. After mixing, the microspheres were incubated for 20 min in the dark at 25°C at 250 rpm lateral agitation. The activated microspheres were subsequently washed twice with coupling buffer, after which 200 μl of antigen was diluted in the coupling buffer at the chosen concentration. These suspensions were incubated at 250 rpm horizontal agitation for 2 hours at 37°C. After incubation, the microspheres were washed three times with washing buffer (PBS, containing 1% BSA, 0.05% Tween 20). The final microsphere suspensions were counted (Beckman Coulter Z3, Kendall-FL, USA) and adjusted to a concentration of 4 x 10^4^ microspheres/ml in storage buffer (PBS containing 1% BSA and 0.02% sodium azide) and stored, protected from light at 2–8°C in low binding tubes (#0030 108.116, Eppendorf, Hamburg, Germany) for 24 hours. The buffer conditions and antigen concentration for coupling were as follows: TpN17 and TpN15 in PBS pH 7.2 at 25 μg/ml, TpN47 in PBS pH 7.2 at 50 μg/ml and TmpA in PBS pH 7.2 at 80 μg/ml. These conditions correspond to the higher signal to noise ratios and the best ROC curve (definition in S1 Text) after assaying them against a set of positive and negative serum samples. The LMA immunoassays were performed using serum samples diluted to 1:100 in assay buffer (PBS containing 1% BSA, 0.05% Tween 20). Fifty μl of microsphere suspension (~2,500) and 50 μl of diluted serum were mixed in each well of a 96-well plate and incubated for 15 min in the dark at 37°C with horizontal rotation at 600 rpm. The microspheres were then washed twice with 100 μl of wash buffer in Hydroflex plate washer with magnetic plate support (TECAN, Durham-NC, USA). Goat anti-human IgG conjugated to phycoerythrin (GTIG-001, Moss substrates, Pasadena-MD, USA) diluted 1:1,000 in assay buffer was added and microspheres were incubated for 15 min in the dark at 37°C with horizontal rotation at 600 rpm. The microspheres were washed twice with 200 μl of wash buffer and once with Sheath Fluid 1x (Luminex Corp, Austin-TX, USA). The results, expressed as median fluorescence intensity (MFI), was determined with a Luminex 200 device.

### ELISA

The optimal antigen coating and antibody-enzyme conjugate (HRP) dilutions, as well as serum dilutions, were determined by checkerboard titration. Final conditions were selected based on maximum divergence in average optical density (OD) values by comparing values of positive and negative samples plus three standard deviations (SD). Results were considered acceptable when positive samples presented an average OD above 1.0 and negative samples were below 0.25. After optimization, the recombinant proteins were added to transparent flat-bottom polystyrene microplates (Microtest Plate 96 Well, F—Sarstedt, Germany) at 100 ng (TpN17 and TpN47) and 200 ng (TmpA) per well in coating buffer (0.05 M carbonate-bicarbonate, pH 9.6). TpN15 protein was not available for testing using indirect ELISA. The microplates were then blocked with WellChampion™ (Ken-En-Tec Diagnostics A/S, Taastrup, Denmark) synthetic blocking buffer according to the manufacturer’s instructions. Serum samples (100 μl) were loaded at 1:25 in 0.05 M phosphate-buffered saline—PBS (pH 7.4). Following incubation for 60 min at 37°C, the microplates were washed in 0.05% Tween-20 in PBS buffer (PBS-T) to remove any unbound antibodies. HRP-conjugated goat anti-human IgG (Bio-Manguinhos, Fiocruz/RJ, Brazil) (100 μl/well) was diluted at 1:10.000 (TmpA) or 1:20.000 (TpN17 and TpN47) in PBS and added to each well, followed by incubation for 30 min at 37°C and washing with PBS-T. For detection of the immunocomplexes, 100 μl of TMB Plus solution (tetramethyl-benzidine; Ken-En-Tec Diagnostics A/S, Taastrup, Denmark) was added to each well. Following 15 min of incubation at RT in the dark, the reaction was stopped with 50 μl 0.3 M H_2_SO_4_, and absorbance was measured at 450 nm in a microplate spectrophotometer (SPECTRAmax 340PC^®^, California, USA).

### Within-run imprecision assessment

Within-run imprecision (or repeatability), using ELISA as a diagnostic platform, was estimated by evaluating a set of sera (22 *T*. *pallidum*-positive and 10 negative samples randomly chosen) in quadruplicate within the same run. The results were analyzed considering arithmetic means of the RI, as well as standard deviation and coefficient of variation determinations.

### Statistical analysis

Statistical tests were performed using scatter computer graphic software (GraphPad Prism version 7, San Diego-CA, USA). The variables were analyzed through descriptive measures such as arithmetic and geometric means, standard deviation (SD) and coefficient of variation (CV). The geometric means were calculated with a confidence interval of 95% CI. Shapiro-Wilk test was applied for testing the data normality. When rejecting the null hypothesis, the Wilcoxon-Mann-Whitney or Kruskal-Wallis test was used. When the normality of the data was confirmed, the Student's T-test was employed. All conclusions were taken at a significance level of p <0.05. The absence of overlapping of the 95% CI values was considered as statistical significance. Cut-off (CO) values were established based on the area under ROC curve analysis. The results were normalized by calculating the reactivity index (RI) that denotes the ratio between the OD of the samples and the CO. Samples that resulted in RI ≥ 1.0 were considered positive. If a sample's RI value fell within ± 10% of 1.0, it was classified as inconclusive (grey zone). To assess global accuracy for each antigen, areas under the ROC curve (AUC) were calculated and interpreted as outstanding (1.0), elevated (0.82–0.99), moderate (0.62–0.81) or low (0.51–0.61) [29]. LMA and ELISA performance parameters were determined and compared regarding sensitivity (Sen), specificity (Spe) and accuracy (acc). The agreement strength between the standard tests and the LMA or ELISA was established by Cohen's kappa (κ) analysis, which was interpreted as follows: 1.0 ≤ κ ≥ 0.81 (almost perfect agreement), 0.80 ≤ κ ≥ 0.61 (substantial agreement), 0.60 ≤ κ ≥ 0.41 (moderate agreement), 0.40 ≤ κ ≥ 0.21 (fair agreement), 0.20 ≤ κ ≥ 0 (slight agreement), and k = 0 (poor agreement) [30]. A checklist (S1 Table) are provided according to the Standards for the Reporting of Diagnostic accuracy studies (STARD) guidelines. A list of abbreviation and definition for ROC curve, AUC, 95%CI, RI, sensitivity, specificity, accuracy, CV, SD and CO is available in S1 Text.

## Results

### Acquisition of recombinant proteins

**Fig 1:**
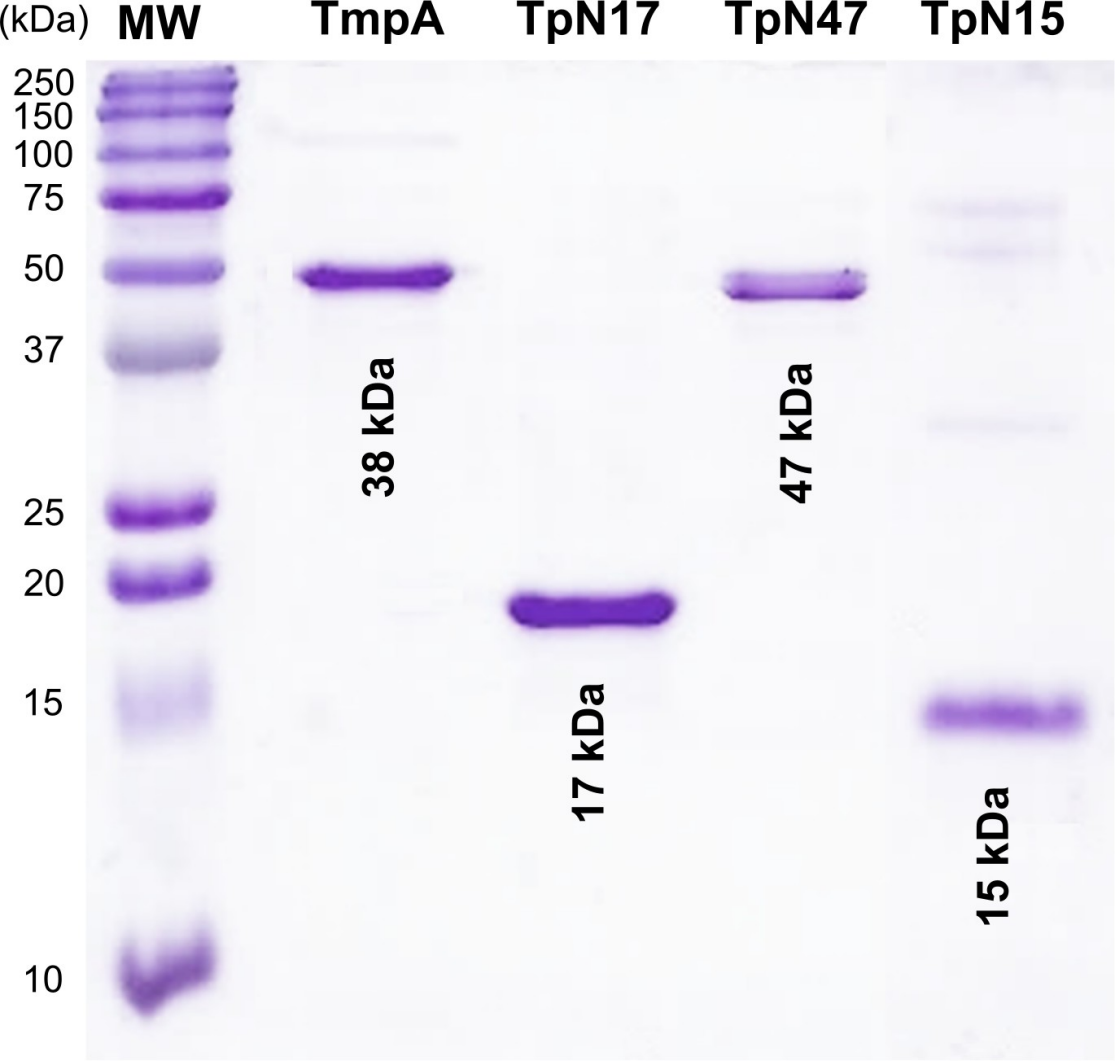
SDS-PAGE stained with coomassie brilliant blue. Recombinant proteins: 1 μg of each antigen was loaded per lane. Antigens are identified at the top of each lane. kDa: Kilodaltons; MW: molecular weight.

After cell-disruption and centrifugation, recombinant proteins were recovered from the supernatant, with purity estimated at over 95% (Fig 1; raw image in S1 Raw Image). As satisfactory protein purity was not obtained through one-step purification, two chromatographic steps were necessary as described above. Satisfactory recovery yield ranged from 1.7 to 2 mg/l of culture volume, with TpN47 being the least productive. The recombinant proteins presented predicted molecular masses of 17, 47, 49 and 15 kDa for TpN17, TpN47, TmpA and TpN15, respectively.

### Sera characterization

**Fig 2:**
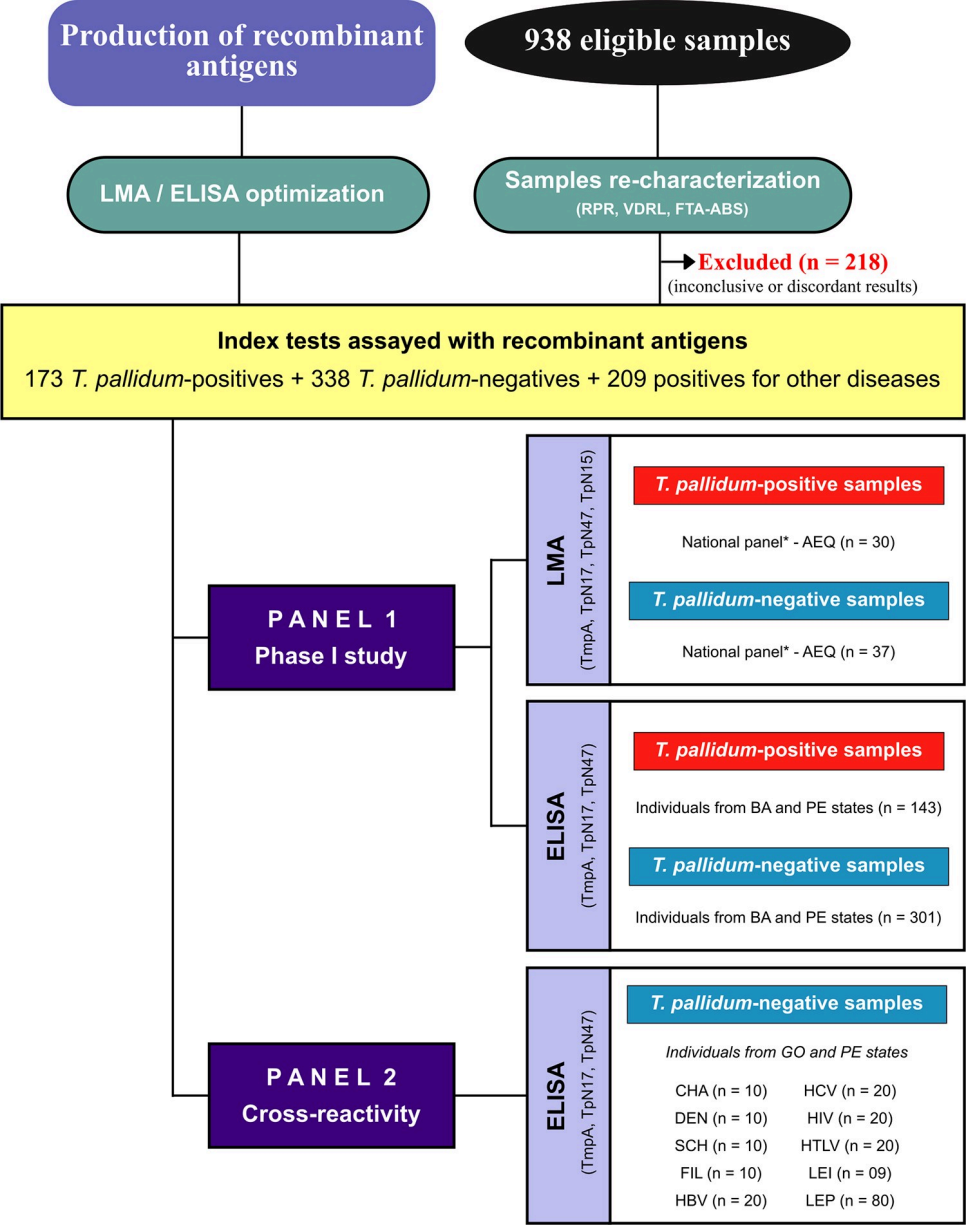
Flowchart detailing diagnostic performance evaluations of recombinant antigens used in the serological diagnosis of syphilis. Reference Standards Tests: RPR (Rapid Plasma Reagin), USR (Unheated Serum Reagin) and IIFT (indirect immunofluorescence—IgG). BA (Bahia); GO (Goiás); PE (Pernambuco); CHA (chronic Chagas disease); DEN (dengue), SCH (chronic schistosomiasis), FIL (filariasis); HBV (hepatitis B); HCV (hepatitis C); HIV (human immunodeficiency virus); HTLV (human T cell lymphotropic virus); LEI (leishmaniasis); LEP (leptospirosis); LMA (Liquid microarray); ELISA (enzyme-linked immunosorbent assay).

A total of 938 serum samples were employed in the present study (Fig 2). All samples were initially re-assayed for non-specific *T*. *pallidum* antibodies assays or anti-*T*. *pallidum u*sing non-treponemal or treponemal tests, respectively. Any samples that returned discordant or inconclusive results were excluded (n = 218; ~ 23%). The present study was performed using two distinct serological panels. Panel 1 was composed of sera from AEQ-panel (LMA analysis; n = 67) and samples from patients living in Bahia and Pernambuco states (ELISA analysis; n = 444), whereas panel 2 was formed by sera from individuals infected with unrelated diseases (n = 209).

### LMA performance

**Fig 3:**
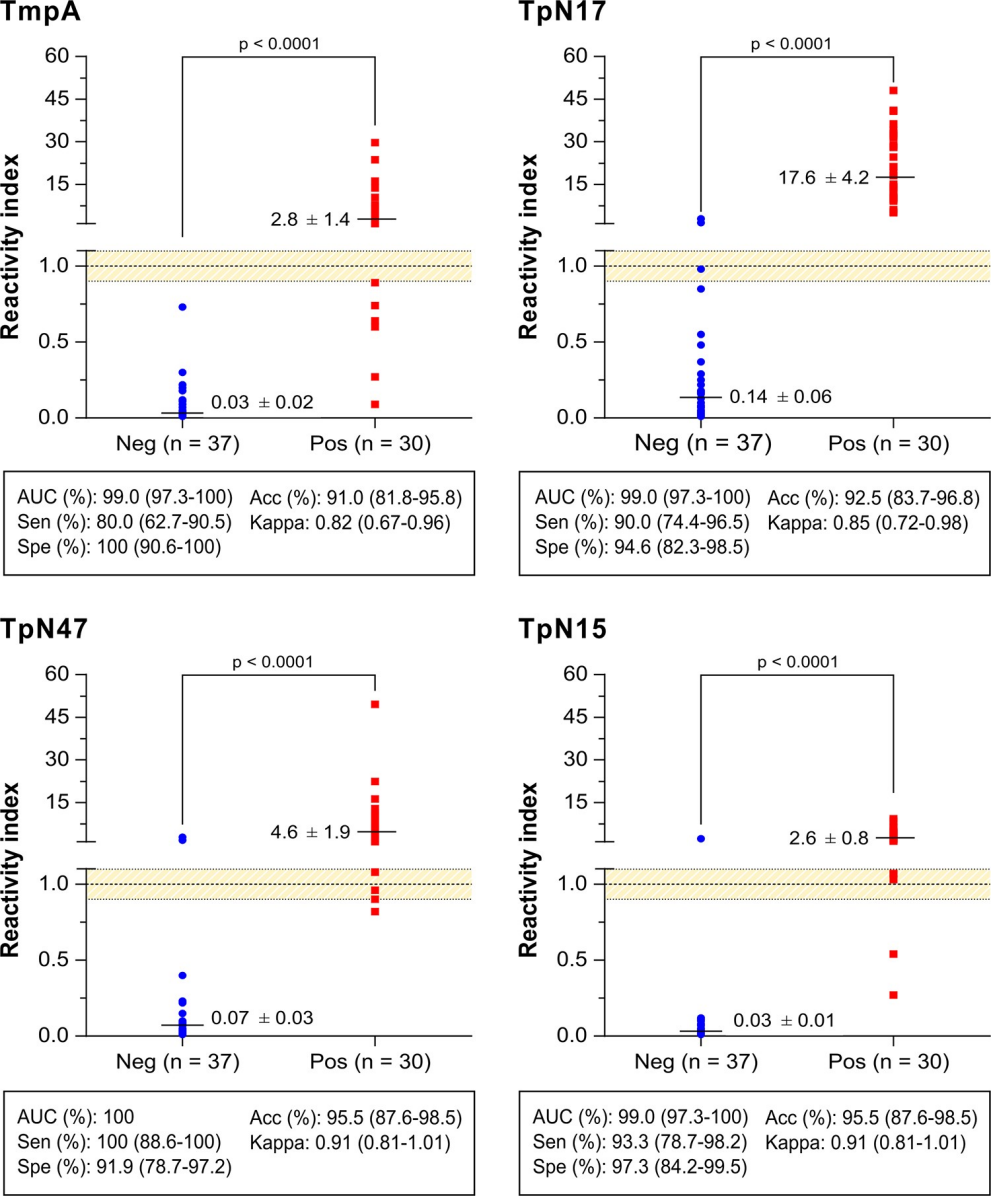
Anti-*Treponema pallidum* IgG level in serum samples from AEQ panel by liquid microarray. The cut-off is set at the reactivity index value = 1.0 and the orange shadowed area represents the grey zone (RI = 1.0 ± 0.10). Horizontal lines and numbers for each result group represent the geometric means (± 95% CI). Acc (Accuracy); AEQ (Blood Bank Screening and Hemotherapy Quality Control Program); AUC (Area Under Curve); Neg (negative); Pos (positive); Sen (Sensitivity); Spe (Specificity).

Initially, 67 serum samples from the AEQ panel were assayed by LMA using four *T*. *pallidum*-recombinant proteins (Fig 3; S2 Table). Based on the total number of samples, the area under ROC (AUC) varied from 99.0% for TmpA, TpN15 and TpN17 proteins to 100% for TpN47, demonstrating excellent diagnostic accuracy for all *T*. *pallidum*-recombinant proteins. In the LMA analyses, the sensitivity score yielded values higher than 90% for TpN17 and TpN15 and 100% for TpN47. TmpA yielded the lowest sensitivity value (80.0%). Global analysis of negative serum samples revealed that the lowest and highest specificity values were presented by TpN47 (91.9%) and by TmpA (100%), respectively. The accuracy of the *T*. *pallidum*-recombinant proteins was also assessed. As shown in Fig 3, TpN47 and TpN15 revealed the best accuracy (95.5%) among the four recombinant proteins assayed, while accuracy values for TmpA and TpN17 were 91.0% and 92.5%, respectively. The strength of agreement between the LMA results and the reference tests was almost perfect for all proteins. At 95% CI, the sensitivity, specificity and accuracy scores showed no difference among the antigens tested by LMA. For positive samples, the TpN17 antigen displayed the highest RI (17.6 ± 4.2) followed by TpN47 (4.6 ± 1.9), TmpA (2.8 ± 1.4) and TpN15 (2.6 ± 0.8). With respect to the negative samples, RI values were lower than 0.15 for all *T*. *pallidum*-recombinant proteins. Considering the RI analysis, discrimination between positive and negative samples was highly significant (p<0.0001). Based on the AUC and accuracy results described above, all four proteins were eligible for phase 1 study using the ELISA platform except TpN15, as there was not enough protein to continue the study.

### ELISA performance

**Fig 4:**
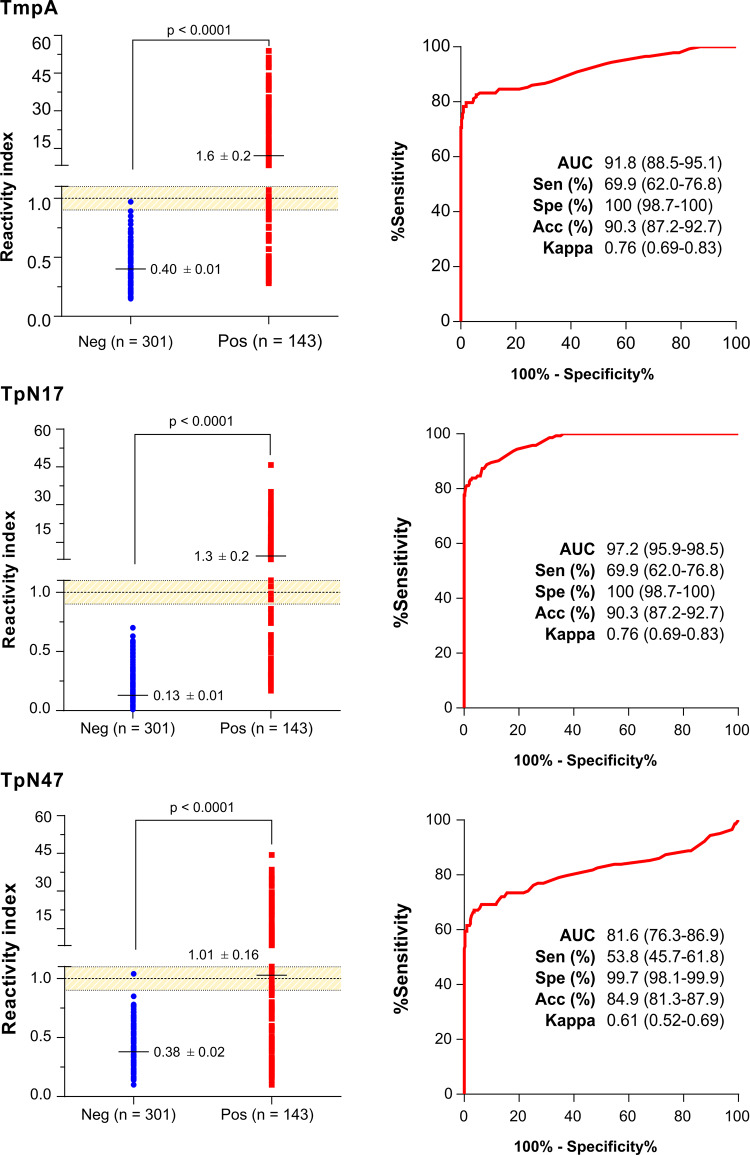
Reactivity index and diagnostic performance parameters obtained with *Treponema pallidum*-positive and negative serum samples. The cut-off is set at the reactivity index value = 1.0 and the shadowed area represents the grey zone (RI = 1.0 ± 0.10). Horizontal lines and numbers for each result group represent the geometric means (± 95% CI). AUC (Area Under Curve); Sen (Sensitivity); Spe (Specificity); Neg (negative); Pos (positive).

Considering the pre-established criteria (OD < 0.25 for negative samples and OD > 1.00 for positive samples) and the highest difference between the arithmetic mean OD for positive and negative plus three standard deviations (DP), antigen concentration were established at 100 ng (TpN17 and TpN47) and 200 ng (TmpA), antibody-enzyme conjugate diluted at 1:10.000 (TmpA) and 1:20.000 (TpN17 and TpN47), and serum samples diluted at 1:25. ROC curves were generated from 143 *T*. *pallidum*-positive samples and 301 negative samples assayed by ELISA (Fig 4; S3 Table). The AUC values were 91.8% for TmpA, 97.2% for TpN17 and 81.6% for TpN47. Considering these values, *T*. *pallidum*-recombinant proteins exhibited excellent (TmpA and TpN17) and good (TpN47) discrimination power and high diagnostic values. The TmpA and TpN17 *T*. *pallidum*-recombinant proteins produced a sensitivity of 69.9% followed by TpN47 (53.8%). The differences in sensitivity are statistically significant for the values obtained for TmpA and TpN17 relative to the values obtained for TpN47. The maximum value of specificity was obtained with TmpA and TpN17 (100%). Assays with TpN47 exhibited a specificity value of 99.7% (Fig 4). Accuracy achieved the maximum value when the samples were assayed with TmpA and TpN17 (90.3%). A lower value was found for TpN47. However, at 95% CI, accuracy scores showed no difference among the three antigens. Differences in sensitivity, specificity and accuracy between serological panels from Bahia and Pernambuco states are not statistically significant (data not shown). Kappa values showed substantial (TmpA and TpN17) and moderate (TpN47) agreement with the reference tests. Considering the inconclusive zone (RI = 1.0 ± 10%), few positive samples yielded inconclusive results, being 14 (4.67%) inconclusive for TpN17 testing, 12 (4%) for TmpA and 11 (3.67%) and for TpN47. No negative sample fell inside the inconclusive space when tested using TpN17. However, one negative sample presented inconclusive results when assayed with TmpA and TpN47. With respect to the *T*. *pallidum*-positive samples, TmpA showed the highest RI value (1.62 ± 0.38), followed by TpN17 (1.28 ± 0.35) and TpN47 (1.02 ± 0.41). No significant difference was observed between the three proteins. For *T*. *pallidum-*negative samples, RI value was lower for TpN17 (0.13 ± 0.32), followed by TpN47 (0.38 ± 0.14) and TmpA (0.40 ± 1.6). Similarly, to positive samples, no significant difference was observed between the three proteins, except when TpN47 was compared to TmpA (p = 1.005).

### Within-run imprecision assessment

**Fig 5:**
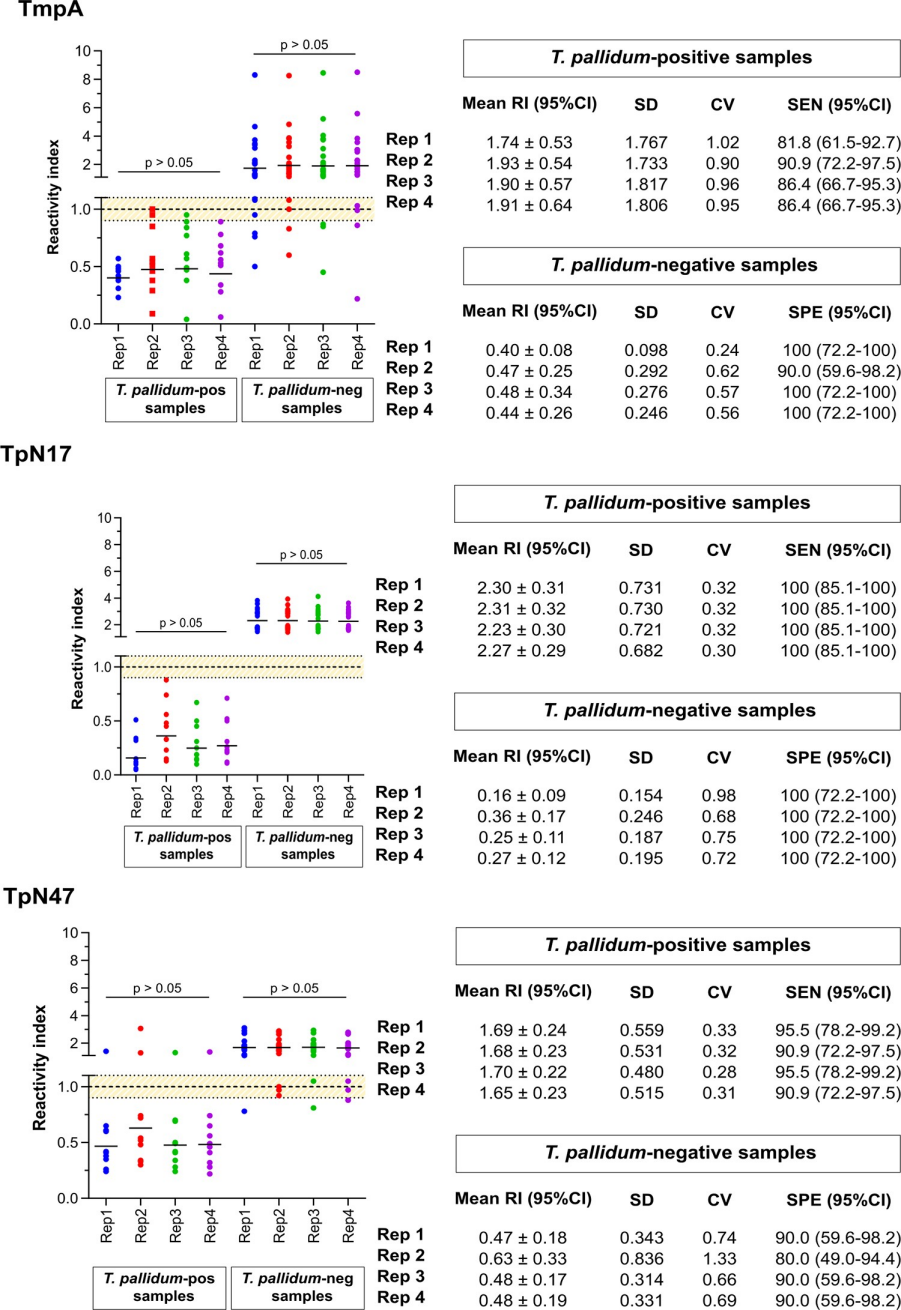
Reactivity index and coefficient of variation in the within-run imprecision assessment. The cut-off is set at the reactivity index value = 1.0 and the shadowed area represents the grey zone (RI = 1.0 ± 0.10). Horizontal lines and numbers for each result group represent the geometric means. CI (confidence interval); CV (coefficient of variation); Neg (negative); Pos (positive); SEN (Sensitivity); SPE (Specificity); Rep (replicate); RI (reactivity index); SD (Standard Deviation).

The assays were performed using 22 *T*. *pallidum*-positive samples and 10 negative samples. Each sample was analyzed in quadruplicate, named replicate 1, replicate 2, replicate 3 and replicate 4 (Fig 5; S4 Table). According to the performance parameters, each one of the four repetitions reached high sensitivity and specificity values. Performance parameters assessed did not present statistically significant differences among all repetitions for the same serum sample. Similarly, no statistical difference in reactivity index was observed considering the 95% CI values overlapping of each measurement. The coefficient of variation (CV) was also considered in the within-run imprecision assessment, which was within a 10% range for each antigen, indicating adequate reproducibility.

### Assessment of cross-reactivity

#### With non-bacterial unrelated infectious diseases

**Fig 6:**
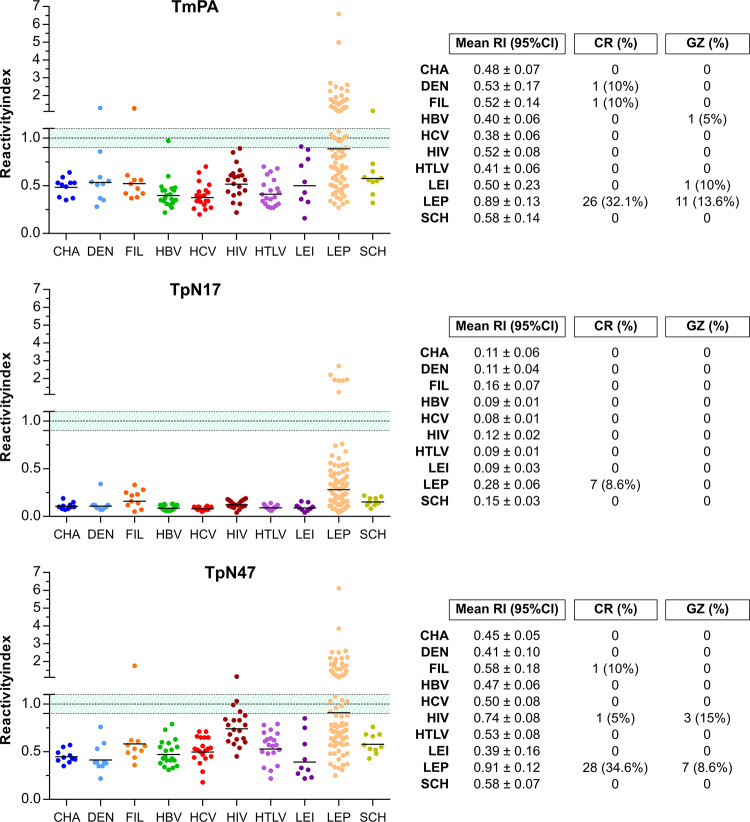
Analysis of the cross-reactivity of the *Treponema pallidum*-recombinant proteins to sera from non-bacterial unrelated diseases. The cut-off value is reactivity index = 1.0 and the shadowed area represents the grey zone (RI = 1.0 ± 0.10). CHA (chronic Chagas disease); DEN (dengue), SCH (chronic schistosomiasis), FIL (filariasis); HBV (hepatitis B); HCV (hepatitis C); HIV (human immunodeficiency virus); HTLV (human T cell lymphotropic virus); LEI (leishmaniasis).

The panel composed of 129 serum samples was employed to assess cross-reactivity of antibodies produced against several infectious parasitic and viral diseases. As shown in Fig 6 (S5 Table), no cross-reaction was observed when serum samples were assayed using TpN17. The incidence of cross-reactivity using TmpA and TpN47 antigens was negligible; only three (dengue, filariasis and schistosomiasis) and two (filariasis and HIV) positive samples produced a false-positive result, respectively. Considering the grey zone (RI values of 1.0 ± 0.10), relatively few samples were considered inconclusive: one positive sample for HBV and *Leishmania* ssp. in the TmpA assay, 3 HIV-positive samples in the TpN47 assay. No sample was inconclusive when assayed using the TpN17 antigen and all other samples produced relatively low RI intensities (<0.80).

#### With leptospirosis

**Fig 7:**
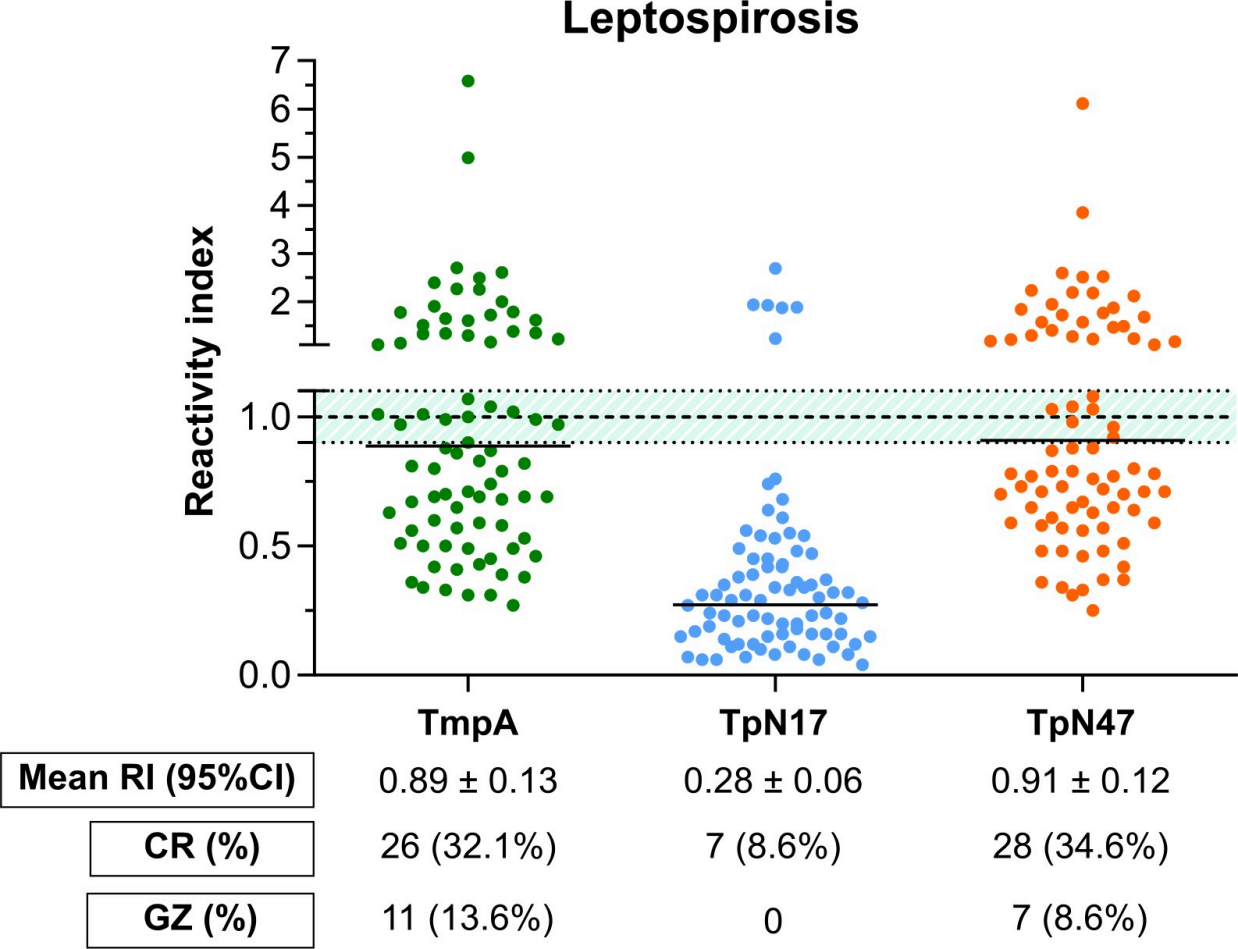
Analysis of the cross-reactivity of the *Treponema pallidum*-recombinant proteins to sera from leptospirosis patients. The cut-off value is reactivity index = 1.0 and the shadowed area represents the grey zone (RI = 1.0 ± 0.10). LEP (leptospirosis).

The panel composed of 80 serum samples was employed to assess cross-reactivity of antibodies produced against leptospirosis. As shown in Fig 7 (individual data points are available in the S5 Table), a surprisingly high number of leptospirosis-positive samples assayed using TmpA (32.1%) and TpN47 (34.6%) presented positive results. TpN17 also presented undesirable results when leptospirosis-positive samples were assayed, but to a lesser extent (8.6%). Considering the grey zone (RI values of 1.0 ± 0.10), 13.6% and 8.6% of leptospirosis-positive samples fell inside the inconclusive space when assayed using TmpA and TpN47, respectively. No sample was inconclusive when assayed using the TpN17 antigen.

### Conservation of the *T*. *pallidum* TpN17, TpN47 and TmpA proteins

An initial hypothesis to explain the high cross-reactivity results of the *T*. *pallidum* antigens observed in serum samples of leptospirosis patients can be the conservation of the protein sequences among bacteria. Therefore, we have evaluated amino acid sequence conservation of these antigens using the BLASTP and PSI-BLAST tools and searching the Genbank database (https://blast.ncbi.nlm.nih.gov/Blast.cgi). TpN17 belongs to the lipoprotein NlpE protein family and TpN17 homologues are found in a wide range of bacteria including pathogenic and enteric species. For TpN17, amino acid identity was found in the range of 34–39% and similarity in the 49–57% range. A search limited to Leptospiracaea identified four proteins showing 35–40% identity over a segment of 50–60 amino acids (*Leptonema illini* putative glycolipid-binding domain-containing protein, sequence ID: WP_002775996.1; *Leptospira broomii* 4Fe-4S binding protein sequence ID: WP_010568632.1; *Leptospira fletcheri* type I 3-dehydroquinate dehydratase, sequence ID: WP_135767117.1; *Leptospira inadai* 4Fe-4S binding protein, sequence ID: WP_020988179.1). TpN47 homologues were also found pathogenic and enteric bacterial species although the number of species in the Genbank containing TpN47 homologues seems to be smaller than for TpN17. TpN47 homologues share approximately 27–28% amino acid identity and 40–42% similarity. A search limited to Leptospiracaea identified three proteins showing ~30% identity over a segment of 109 amino acids (alpha/beta hydrolase, sequence ID: WP_108927162.1 and PGAP1-like domain protein, sequence ID: GBF37386.1 in *Leptospira johnsonii* and TetR/AcrR family transcriptional regulator, sequence ID: WP_109022509.1 in *Leptospira kobayashii*). Genbank searches returned TmpA homologues only for species from the genus *Treponema* with amino acid identity and similarity in the range of 30% and 50%, respectively. A search for TmpA homologues limited to Leptospiracaea did not retrieve any sequence.

## Discussion

Here, we assessed the diagnostic performance of four recombinant proteins (TpN17, TmpA and TpN47) for detecting specific anti-*T*. *pallidum* antibodies in serum of syphilis-positive individuals. Initially, the ability of recombinant antigens to differentiate *T*. *pallidum*-positive from negative samples was assessed by liquid microarray (LMA). All four proteins showed AUC and accuracy values above 99% and 91%, respectively (Fig 3). Similar results were described in other studies [31–33]. In fact, studies using the BioPlex 2200 syphilis IgG assay revealed a sensitivity from 95.8% to 100% and specificity from 89.7% to 99.4% for the proteins TpN15, TpN17 and TpN47 assayed together [31–33]. On the other hand, BioPlex 2200 syphilis IgM assay showed a sensitivity of 80% and specificity of 97.9% [32]. The diagnostic performance of TpN17 and TmpA was also evaluated in multiplex bead assay for the detection of specific-*T*. *pallidum* IgG antibodies using TPPHA or RPR as reference tests. Compared to TPPA/TPHA, TpN17 and TmpA revealed a sensitivity of 90.1% and 67.5%, respectively, while the specificity values were 97.6% and 99.2%, respectively. In the case of RPR as reference test, sensitivity and specificity values were 94.8% and 92.7% for TpN17 and 79.9% and 98.2% for TmpA, respectively [34]. The results this study highlighted the higher efficiency of TpN17 and TpN47 for the detection of syphilis infection as compared to TmpA. Based on the AUC and accuracy values, our results are consistent with previous studies but due to the non-availability of TpN15 in the study, only three proteins were assessed for the phase I study by ELISA.

Similarly to LMA results, ELISA also showed high AUC (81.6% to 97.2%) and accuracy values (84.9% to 90.3%) for TpN17, TmpA and TpN47. No difference in performance was observed between samples from distinct Brazilian endemic settings (Bahia and Pernambuco), suggesting that all three antigens can be used regardless of geographical area. Indeed, *T*. *pallidum* strains have a high genomic similarity, ranging from 99.57 to 99.98%, without significant genetic variability capable of influencing diagnostic tests [35]. Related studies have already evaluated the diagnostic performance of recombinant proteins for the diagnosis of syphilis by ELISA but have obtained results divergent from our findings [17, 36–38]. Indeed, studies assessing the performance of TpN17 revealed sensitivities of 84.4% [17], 88.9% [37] and 100% [36, 38]. Regarding specificity, TpN17 was described as 100% specific [17, 36–38]. In the present study, we have found ~70% of sensitivity for this antigen in ELISA analysis. With respect to TmpA, high sensitivity and specificity values of 83.3% and 100% [37] and 89.6% and 100% [36], respectively, have also been reported. Considering the infection clinic stage, the TmpA protein showed a sensitivity of 76% for primary syphilis, 100% for secondary syphilis and 98% for early latent syphilis with specificity values of 100% for all [39]. Finally, TpN47 showed a diagnostic sensitivity of 82.1% [17], 87.8% [36] and 88.9% [37] and specificity of 100% [17, 36, 37]. However, the low sensitivity values showed here were possibly due to the serological panel, whose samples have not been classified according to the stage of infection for each individual [39]. Recombinant antigens may have different antibody reactivities depending on the individual's stage of *T*. *pallidum* infection [38], and when ELISA is used with isolated recombinant antigens, the test was shown to be less sensitive than serological tests with antigenic mixtures [40]. Although not evaluated by the ELISA, TpN15 has already been described with 90.24% [36] and 100% [24] sensitivity and 100% [24, 36] specificity on that same diagnostic platform.

Compared to non-treponemic tests, treponemics perform better and are more useful in reverse screening. This new protocol for the diagnosis of syphilis provides greater confidence in the results because allows diagnoses cases of latent syphilis that may not have been detected by traditional screening, besides in addition to reducing the rates of false positive results associated others infectious disease with hepatitis, mononucleosis, viral pneumonia, chickenpox, measles, malaria, as well as vaccinations, pregnancy and a technical error in the execution of the tests [10]. Studies evaluating the reverse algorithm for syphilis screening have shown its higher sensitivity in comparison with the traditional screening algorithm due to its capacity to detect cases unlikely to be diagnosed by nonspecific tests. Thus, the treponemal antigens used as an antigenic matrix in these tests allow a better performance of the diagnostic platform used [41–46]. Indeed, a study showed that after the introduction of the reverse algorithm the percentage of false positive test results in Brazilian blood donors decreased from 60.7 to 25.6%. Furthermore, the discrepancy between initial chemiluminescence result and subsequent VDRL test result was reduced to 25.9%, as compared with 36.6% before its introduction [47].

Differences in sensitivity between LMA and ELISA may have been influenced by the differences in characteristics inherent in each diagnostic platform used. In LMA, the median fluorescence intensity (MFI) of the detection antibody corresponds to an average of 100 bead readings; that is, a single serum sample is analyzed 100 times per antigen, whereas in ELISA the sample is analyzed only once. This level of accuracy improves the detection limit by LMA assays [48, 49]. Despite the advantages offered by LMA-based technology, the need for a more complex laboratory infrastructure, well-trained workforce, and substantial financial investment [50] did not allow the study to be conducted using this diagnostic methodology alone.

In order to investigate the consistency of the results in the repetition of reactions, the analysis of intraplate reproducibility or repeatability was performed. In the analysis of the three molecules, there were no statistically significant differences in terms of AUC, sensitivity and specificity and RI values. For reproducibility to be acceptable, the coefficient of variation (CV) must be less than 20% [51]. In our case, we found values ≤1% for positive samples and <2.19% for negative samples, indicating that antigen assays have good repeatability. In a study for evaluation of the Elecsys Syphilis Immunoassay (double-antigen sandwich), the analytical performance was also assessed for within-run repeatability. The SD for negative pools (IR < 1.0) ranged from 0.01 to 0.02, and the SD for positive pools (IR ≥ 1.0) ranged from 0.01 to 0.13. All the human serum pools met for the CV expected target, indicating good repeatability [52].

In the present study, cross-reaction was evaluated against serological samples from individuals with unrelated non-bacterial diseases, such as chronic Chagas disease, dengue, hepatitis B, hepatitis C, HIV-1/2, chronic schistosomiasis, HTLV-1/2, filariasis, cutaneous and visceral leishmaniasis and with the bacterial disease leptospirosis. All samples were revaluated for the presence of syphilis coinfection, to rule out the possibility of false positives due to presence of IgG anti-*T*. *pallidum*. Except for leptospirosis for which all proteins showed high cross-reactivity, TpN17 did not false-positive results and a small number of cross-reactive samples were observed for the other two proteins. TmpA false-positive samples were observed for dengue, filariasis and chronic schistosomiasis and TpN47 false-positives were detected for filariasis and HIV-1/2. A previous study using 40 hepatitis B positive samples reported only one false-positive result for TmpA [53]. Conversely, a second study identified two hepatitis B samples as positive for TpN47 and TmpA [24], while a third study reported only 1 hepatitis B and 1 HIV positive sample for TmpA [20]. In all cases, there was no clear hypothesis to explain the cross-reactions found.

With regards to leptospirosis cross-reaction, false-positive results could be due to similarity among proteins of *T*. *pallidum* and *Leptospira interrogans*. A basis on the level of antibody cross-reactivity with antigens from different bacterial species has already been described in previous studies [54–56]. Searches in the Genbank database did not identify any homologue of these three proteins in *Leptospira interrogans*. However, homologues of TpN17 and TpN47 are found in bacterial genomes, including in pathogenic and enteric species, as well as in some other species of the genus *Leptospira*. It is unclear why homologues of these proteins are not found in *Leptospira interrogans* genome sequences. This is most probably due to conservation of the epitopes in proteins across bacteria. The case of TmpA is even more intriguing. The information retrieved from the database using sequence similarity searches identified TmpA homologues only in species from the genus *Treponema*. With this information only, it is not possible to propose a hypothesis for cross-reactivity based on conservation of between *T*. *pallidum* TmpA and other bacterial species. These results cannot then be interpreted only as false-positive, but perhaps as a fact that needs to be further investigated. Two other possible explanations for our findings have been proposed previously. For a possible nonspecific reaction, it is likely that individuals with positive samples for unrelated diseases also had been previously treated syphilis, but antibodies remained circulating in the body [20]. However, we sought to rule out the possibility of using samples that were also positive for syphilis by retesting all serum samples with two reference test and excluding all that were not negative for these two assays. We cannot exclude also the possibility that some false-positive results might be due to the existence of some *Escherichia coli* impurities or epitopes with the recombinant antigen as a result of poor purification, which could promote nonspecific cross-reactions. One such interference caused by nonspecific proteins has already been evaluated in an ELISA using a recombinant antigen and the lack of specificity of the test has been related to protein residues translated from the plasmid vector gene sequence responsible for cross-reactions [20].

The main limitation of this study was the use of two non-treponemal tests as a reference test to recharacterized several samples, which might not have a good correlation with treponemal tests, and the impossibility of using immunofluorescence throughout the serological panel. Thus, the diagnostic performance of recombinant antigens may have been compromised due to the inefficiencies of the reference tests. Another limitation was the impossibility of obtaining clinically well-defined serological samples. Nevertheless, we conclude that despite the low sensitivity values in ELISA, the proteins showed high diagnostic capacity due to the AUC values found. However, an improvement in sensitivity can be achieved when antigenic mixtures are evaluated.

## Supporting information

S1 Table STARD checklist. Standards for the Reporting of Diagnostic Accuracy Studies (STARD) checklist for reporting of studies of diagnostic accuracy.
(DOCX)

S2 Table Reactivity index for by LMA diagnostic performance assessment. (XLSX)

S3 Table Reactivity index for by ELISA diagnostic performance assessment. (XLSX)

S4 Table Within-run imprecision assessment. (XLSX)

S5 Table Reactivity Index for cross-reactivity assessment. (XLSX)

S1 Text A list of abbreviation and definition for ROC curve, AUC, 95%CI, RI, sensitivity, specificity, accuracy, CV, SD and CO. (PDF)

S1 Raw Image SDS-PAGE stained with coomassie brilliant blue. Recombinant proteins: 1 μg of each antigen was loaded per lane. Antigens are identified below each lane. Different lots of TpN17 and TpN15 were evaluated. “X” indicates lanes not included in the final Fig 1. kDa: Kilodaltons.
(TIF)
