# Correction: Performance of *Treponema pallidum* recombinant proteins in the serological diagnosis of syphilis

**DOI:** 10.1371/journal.pone.0242386

**Published:** 2020-11-10

**Authors:** Ângelo Antônio Oliveira Silva, Ueriton Dias de Oliveira, Larissa de Carvalho Medrado Vasconcelos, Leonardo Foti, Leonardo Maia Leony, Ramona Tavares Daltro, Amanda Leitolis, Fernanda Washington de Mendonça Lima, Marco Aurélio Krieger, Nilson Ivo Tonin Zanchin, Fred Luciano Neves Santos

The Funding Statement is incorrect. The correct Funding Statement is as follows: This work was supported by the Coordination of Superior Level Staff Improvement-Brazil (CAPES)—Finance Code 001 and Research Support Foundation of the State of Bahia (FAPESB). Nilson I. T. Zanchin and Marco A Krieger are research fellows of National Council for Scientific and Technological Development (CNPq).
