# Supplementary material for: Performance of Treponema pallidum recombinant proteins in the serological diagnosis of syphilis
Source: PLoS One. 2020 Jun 18;15(6):e0234043. doi: 10.1371/journal.pone.0234043 (PMC7302711; doi:10.1371/journal.pone.0234043)
Supplement: S1 Text — (PDF) [file pone.0234043.s006.pdf]

S1 Text - List of abbreviations and definitions

ROC (Receiver Operating Characteristic): A powerful tool for measuring diagnostic test performance in medicine. This analysis employs a robust graphic method to study variations in sensibility and specificity using different cut-off values;

AUC (Area Under the ROC Curve): An index of the discriminating power of a diagnostic test, which can be determined through numerical methods, such as the trapezoidal rule, statistical methods such, as the Wilcoxon-Mann-Whitney test, or maximum likelihood estimations;

95%CI (95% Confidence Intervals): Range of values reflecting an almost 95% degree of certainty that the true value is contained within the range. CI values provide an estimated range that is highly likely to include an unknown population parameter, with the estimated range being calculated from a given set of sample data;

RI (reactivity index): A measure of the optical density of a sample divided by the cut-off value used to normalize the data and standardize results in order to avoid variations inherent in each microplate;

Sensitivity: The probability of a result being positive by the test among the serologically positive samples, thus expressing the total number of correct responses regarding the true number of positive samples;

Specificity: The probability of a result being negative by the test among the serologically negative samples, thus expressing the total of correctly negative tests with respect to the total number of true negative samples;

Accuracy: The ability of a test to correctly identify individuals with a particular disease and to exclude those who do not have the disease. This can be estimated by comparing the results of the index test with the results of the reference standard. When there is a single target condition for dichotomous tests, the accuracy of a diagnostic test is usually calculated by the proportion of truly positive individuals to those who are truly negative in relation to false positives and false negatives;

CV (Coefficient Variation): Ratio of the standard deviation to the mean. The higher the coefficient of variation, the greater the level of dispersion around the mean. The lower the value of the coefficient of variation, the more precise the estimate. This is generally expressed as a percentage. Without units, it allows for comparison between distributions of values whose scales of measurement are not comparable;

SD (Standard Deviation): The standard deviation is a summary measure of the differences of each observation from the mean or the amount of variation or dispersion within a set of values. A low standard deviation indicates that values tend to approximate the mean of the set, while a high standard deviation indicates that values are spread out over a wider range;

CO (Cut-off): Estimated value of a test used to discriminate uninfected individuals from infected individuals.
