# Supplementary figures and images for: Performance of Treponema pallidum recombinant proteins in the serological diagnosis of syphilis
Source: PLoS One. 2020 Jun 18;15(6):e0234043. doi: 10.1371/journal.pone.0234043 (PMC7302711; doi:10.1371/journal.pone.0234043)

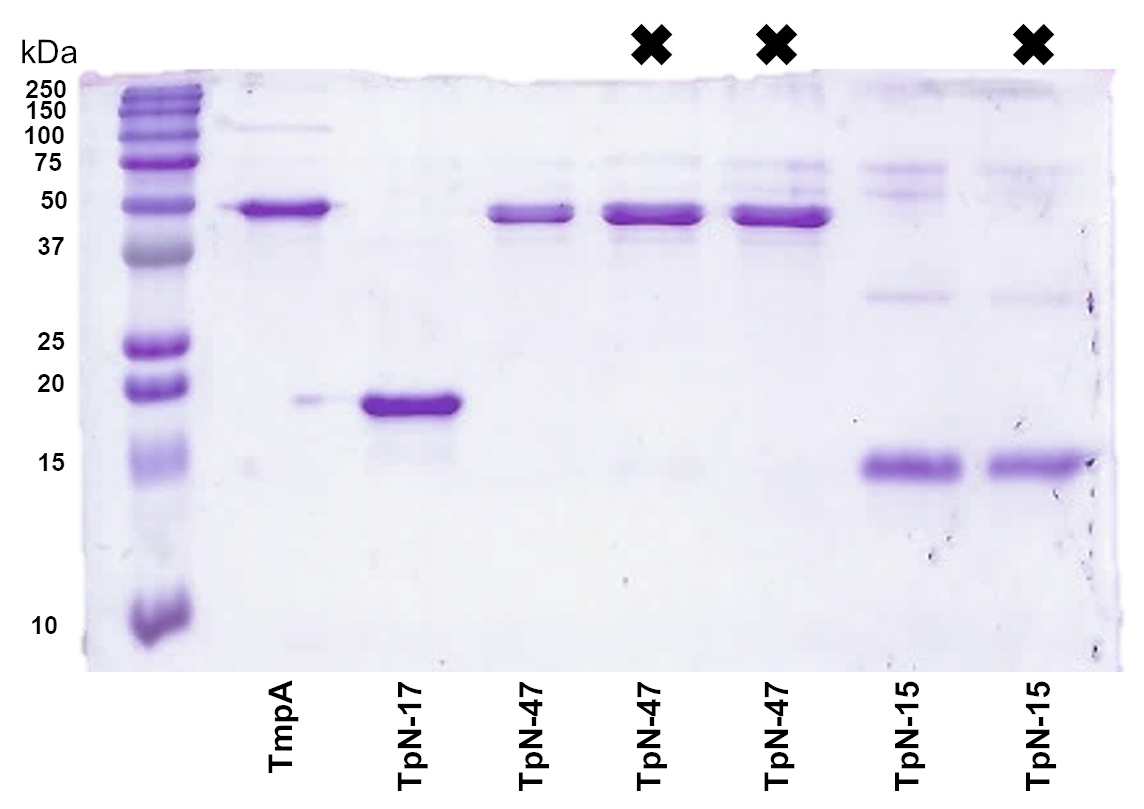

Supplement: S1 Raw Image — Recombinant proteins: 1 μg of each antigen was loaded per lane. Antigens are identified below each lane. Different lots of TpN17 and TpN15 were evaluated. “X” indicates lanes not included in the final Fig 1. kDa: Kilodaltons. (TIF) [file pone.0234043.s007.tif]
